# The Knee injury and Osteoarthritis Outcome Score (KOOS) for lateral tibial plateau fractures– relevance, reliability and responsiveness

**DOI:** 10.1007/s00068-024-02607-7

**Published:** 2024-08-07

**Authors:** Jens Traerup, Peter Larsen, Rasmus Elsøe

**Affiliations:** 1 Department of Orthopaedic Surgery, Aalborg University Hospital, Aalborg, Denmark; 2 Department of Occupational Therapy and Physiotherapy, Aalborg University Hospital, Aalborg, Denmark

**Keywords:** KOOS, Validity, Reliability, Responsiveness, Lateral tibia plateau fracture, Minimal clinically important difference (MCID)

## Abstract

**Background:**

This study aimed to evaluate the patient-reported relevance, test-retest reliability, and responsiveness for each of the five KOOS subscales in patients with lateral tibial plateau fractures.

**Methods:**

Adult patients with surgically treated lateral tibial plateau fractures (AO 41B) were included. The primary outcome measure was the KOOS subscales: Pain, Symptoms, Activity of Daily Living (ADL), Sport and Recreational Activities (Sport/rec), and kne-related Quality of Life (QOL). The KOOS was repeated at 14 and 15 days, six weeks, and 6 and 12 months. Content validity was partly evaluated by patients ranking the relevance of all the items in the KOOS, test-retest reliability by an interclass correlation coefficient, and responsiveness by effect size and based on 3 pre-defined hypotheses related the the global rating of change.

**Results:**

Forty-one patients with a mean age of 54.8 years (ranging from 21 to 81 years) were included. The results showed an acceptable relevance of all the KOOS subscales. The test-retest reliability was moderate to high for all five subscales, with an interclass-correlation coefficient ranging from 0.6 to 0.9. At the 6- and 12-month follow-ups, the responsiveness showed large effect sizes for all the KOOS subscales, ranging from 0.9 to 2.1. Moderate to high correlations (*r* ≥ 0.4)was observed for the predefine hypotheses.

**Conclusion:**

The KOOS questionnaire showed acceptable relevance, high test-retest reliability and acceptable responsivness within one year following a lateral tibial plateau fracture. More research is needed for further validation of psychometric properties of KOOS for patients with lateral tibial plateau fractures.

## Introduction

Although fractures of the lateral tibial plateau are relatively rare in the orthopaedics trauma departments, the fractures remain a surgical challenge due to the complexity of fracture patterns and associated soft tissue injuries [1, 2]. The functional recovery from a lateral tibial plateau fracture may be prolonged, and persistent long-term musculoskeletal difficulties such as knee pain, knee stiffness, limitations in climbing stairs, and participation in sports are common [1, 3–5].

Generic and knee-specific patient-reported outcome measures (PROMs) are considered important instruments to measure the trajectory of functional recovery and quality of life (QOL) in patients following lateral tibial plateau fractures [1, 6]. Unlike generic PROMs, the body-region-specific PROMs may be more sensitive to capture the knee-specific conditions following a lateral tibial plateau fracture [7, 8]. Several generic and knee-specific PROMs have been used to investigate recovery in patients with tibial plateau fractures [6, 9, 10]. However, evidence is lacking in psychometric properties such as validity, reliability and responsiveness for PROMs in patients with lateral tibial plateau fractures.

The Knee Injury and Osteoarthritis Outcome Score (KOOS) is a knee-specific and commonly used instrument to measure the trajectory of functional recovery and quality of life (QOL) in patients following tibial plateau fractures [1, 11–15]. Psychometric properties of the KOOS are well established for several injuries; however, evidence is lacking in patients with lateral tibial plateau fractures [16]. Such psychometric properties can improve the interpretation of the KOOS scores in patients with lateral tibial plateau fractures.

This study aimed to evaluate the patient-reported relevance, test-retest reliability, and responsiveness for each of the five KOOS subscales in patients with lateral tibial plateau fractures.

## Patients and methods

### Study design

This study design is a cohort study. Consecutive patients with lateral tibial plateau fractures (AO 41B1, B2, B3) were included [17]. The primary outcome measurement was The Knee Injury and Osteoarthritis Outcome Score (KOOS) [18] at the 12-month follow-up.

The North Denmark Region approved the study (K2023-018), and the Danish Data Protection Agency (ID 2020-069) approved the study. Informed consent forms were obtained from all patients before their participation. The reporting of the study complies with the Strengthening the Reporting of Observational Studies in Epidemiology (STROBE) statement [19].

### Participants

Surgical treated patients from Aalborg University Hospital, Denmark, were included between June 2020 and March 2022. Included were adult patients (> 18 years of age) presenting with a closed lateral tibial plateau fracture (AO-41- B1, B2, B3) [17] Excluded were patients with multi-trauma, bilateral fractures, severe systemic diseases or cancer, pathological fractures and patients without prior gait function. Moreover, patients with cognitive disorders were excluded.

### Outcomes

At the time of inclusion, basic information regarding age, sex, height and weight, mode of injury, and AO classification were obtained. KOOS outcome was obtained at 14 days, 15 days, six weeks, and 6, 12 months postoperatively.

#### Primary outcome - KOOS

The KOOS is a patient-reported knee-specific questionnaire including 42 items in five subscales evaluating Pain, Symptoms, the function of daily living (ADL), Sport and recreation activities (Sport/rec), and kne-related Quality of life (QOL) [18]. The KOOS is available in more than 45 languages [18]. A score between 0 and 100 is calculated for each of the five subscales. A score of 100 indicates the best possible results and 0 the worst outcome. Normative large-scale reference values of the KOOS subscale scores are available [20]. The KOOS is subject to license, and is available from the mapi research trust webpage. Depending on the context of use, a license fee may apply, however it is free available for academic use.

#### Relevance of the KOOS items

Content validity was party evaluated by asking patients about relevance of the KOOS items [21]. At the six-week and 12-month follow-up, all patients were asked to rate the relevance of the 42 items of the KOOS questionnaire. A three-point Likert scale was used to rate the relevance: not relevant = 0, somewhat relevant = 1, and very relevant = 2. The ratings from each of the five subscales (pain, symptoms, ADL, sport/rec and QOL) were summed, and mean scores were given. A mean score of 1 was predefined as a threshold to demonstrate acceptable relevance.

#### Test-retest reliability

The test-retest reliability was evaluated at 14 days and 15 days after surgical treatment. All patients were asked to score the KOOS questionnaire at the first follow-up (14 days) and again following a time period of 24 h (15 days). A 24 h time period between the 2 measuring points was considered optimal as change in pain, functions and medication is rapid within the first weeks following surgery. The interclass correlation coefficients (ICC) and 95% CI were calculated to investigate the test-retest reliability of the five KOOS subscale scores. ICC values were interpreted as 0.0–0.3, low; 0.30–0.70, moderate; and 0.70–1.0, high [22].

#### Responsiveness

The responsiveness was evaluated throughout the 12-month follow-up period by scoring the KOOS questionnaire at 14 days, six weeks, and 6 and 12 months. The effect size was calculated to investigate the responsiveness of the five KOOS subscales. The effect sizes were interpreted according to Liang et al. as 0.2–0.5 small, 0.5–0.8 moderate, and 0.8–1.0 large effect [23].

Furthermore, reponsiveness was evaluated based on 3 pre-defined hypotheses. Hypotheses were developed based from our clinical experience considering the recovery process from a lateral tibial plateau fracture and expected implications on the KOOS subscales. The hypotheses was related to the global rating of change (GROC) model [24]. At the 6 weeks, 6- and 12-month follow-up, patients were asked to think back to the last visit and judge the degree of change in symptoms. A GROC questionnaire including a seven-point Likert scale was used: “much worse,” “worse”, “a little worse,” “the same/ no change,” “a little better,” “better,” and “much better”. Correlations were interpertated as: high ≥ 0.5,moderate 0.5 − 0.3 and low < 0.3 [25].

1. The change between 6 weeks and 6 moths in the KOOS subscales symptoms, ADL, and QOL will have a positive and moderate correlation with the GROC at 6 months.
2. The change between 6 months and 12 moths in the KOOS subscales ADL, sport and QOL will have a positive and moderate correlation with the GROC at 12 months.
3. The change between 14 days and 6 weeks in the KOOS subscales pain and symptoms will have a positive and moderate to correlation with the GROC at 6 weeks.

Due to a difference in the construct of the GROC question and KOOS subscales only moderate correlations were predefined. With a least 75% of results being in accordance with the hypotheses we will report responsiveness acceptable [26].

### Statistics

The sample sizes in studies to validate PROMs vary, and current literature lacks evidence [27]. Based on available littersture reporting meassurment properties for the KOOS and considering the low incidence of lateral tibial plateau fractures, we predefined a sample size of 41 patients to be included in the present study [2, 16].

The means and 95% confidence intervals (CI) are given for continuous data and frequencies. Per centages are given for categorical data. The effect size was calculated as the difference between the means between 14 days, six weeks, and 6 and 12 months divided by the standard deviation (SD) of the same measure at 14 days. Hypotheses testing were computed by a Spearm rank correlation coefficient.

The statistical analysis was performed with Stata (version 17).

## Results

The mean age of patients was 54.8 years (range 21 to 81). The female gender represented 12 patients, and 29 were men. Detailed baseline characteristics are outlined in Table 1.

**Table 1:** Baseline characteristics of the 41 patients

Mean age (range), [years]	54.8 (21-81)
Male / female, [n]	29/12
Mean height (SD) [cm]	171.8 (8.8)
Mean weight (SD), [kg]	78.1 (15.3)
Fracture classification, AO-41B-1 / 2 / 3 [n]	2/11/28

At the follow-up, after 14 days, 41 patients completed the KOOS. At 15 days 41, at six weeks 40, at six months 40 and at 12 months, 36 patients completed the KOOS questionnaire.

### Relevance of the KOOS items

At the 12-month follow-up, the relevance rating ranged from mean 1.3 to 1.6 between the 5 KOOS subscales. Considering the predefined threshold of 1 demonstrating acceptable relevance, results indicated that KOOS appears to be a relevant instrument to capture patient-perceived outcomes following lateral tibial plateau fractures (Table 2).

At the six-week follow-up, four out of the five subscales (Pain, Symptoms, ADL, QOL) demonstrated an acceptable relevance (ranging from mean 1.3 to 1.6). As expected the subscale Sport/Rec was reported with a low degree of relevance at the six-weeks follow-up (mean 0.7). The subscale Sport/Rec included high level activites such as running and jumping, and most patients were recommended to avoid weight bearing on the injured leg during a period of at least 6 weeks following surgery. (See Table 2a and 2b).

**Table 2a:** Relevance and reliability of the KOOS subscales

	KOOS				
	Pain	Symptoms	ADL	Sport/Rec	QOL
Relevance 6 weeks, mean(95%CI)	1.3 (1.2-1.5)	1.5 (1.4-1.7)	1.3 (1.1-1.4)	0.7 (0.4-1.0)	1.6 (1.5-1.8)
Relevance 12 months, mean (95%CI)	1.3 (1.1-1.6)	1.2 (1.1-1.5)	1.3 (1.0-1.5)	1.5 (1.3-1.8)	1.6 (1.4-1.8)
Test/retest reliability, ICC (95%CI)	0.7 (0.5-0.9)	0.6 (0.4-0.7)	0.8 (0.6-0.9)	0.8 (0.7-0.9)	0.8 (0.6-0.9)

**Table 2b:** Relevance of the 42-KOOS items 6 weeks and 12 months, (%)

KOOS items	6 weeks			12months		
	not relevant	somewhat relevant	very relevant	not relevant	somewhat relevant	very relevant
Symptoms						
1	2%	24%	74%	22%	28%	50%
2	7%	27%	66%	17%	26%	57%
3	24%	22%	54%	17%	37%	46%
4	5%	7%	88%	13%	13%	74%
5	10%	19%	71%	13%	25%	62%
6	17%	39%	44%	20%	41%	39%
7	7%	41%	52%	12%	43%	45%
Pain						
8	2%	22%	76%	7%	21%	72%
9	32%	19%	49%	24%	25%	51%
10	10%	22%	68%	18%	24%	58%
11	17%	17%	66%	18%	18%	63%
12	46%	7%	47%	31%	11%	58%
13	39%	10%	51%	27%	13%	60%
14	10%	32%	58%	20%	25%	55%
15	19%	22%	59%	20%	24%	56%
16	32%	27%	41%	29%	24%	47%
ADL						
17	37%	14%	49%	28%	13%	59%
18	34%	12%	54%	25%	15%	60%
19	17%	29%	54%	16%	28%	56%
20	22%	37%	41%	28%	29%	43%
21	44%	15%	41%	29%	20%	51%
22	44%	12%	44%	30%	17%	53%
23	15%	41%	44%%	14%	40%	46%
24	37%	27%	37%	33%	27%	40%
25	7%	39%	54%	20%	33%	47%
26	10%	29%	61%	16%	29%	55%
27	7%	44%	49%	19%	39%	43%
28	2%	37%	61%	15%	29%	56%
29	15%	24%	61%	23%	27%	51%
30	17%	32%	51%	18%	31%	51%
31	10%	22%	68%	19%	26%	55%
32	51%	15%	32%	8%	26%	66%
33	34%	29%	34%	23%	37%	40%
Sport/Rec						
34	54%	15%	32%	11%	17%	63%
35	63%	7%	29%	17%	11%	72%
36	63%	10%	27%	17%	20%	63%
37	56%	12%	32%	11%	17%	72%
38	61%	12%	27%	11%	14%	75%
QOL						
39	7%	19%	73%	9%	17%	74%
40	7%	15%	78%	8%	22%	70%
41	17%	15%	68%	13%	18%	69%
42	7%	20%	73%	10%	22%	68%

### Test-retest reliability

A high test-retest reliability was observed for the subscales pain, ADL, Sport/rec and QOL. The ICC values ranged from 0.7 to 0.8. The subscale Symptoms demonstrated an ICC of 0.6, indicating moderate test-retest reliability. (See Table 2a).

### Responsiveness

Better KOOS mean scores were observed with an increase in time postoperative for all five subscales (Table 3a).

**Table 3a:** Mean KOOS subscale scores

KOOS, mean (SD)	14 days	6 weeks	6 months	12 months
Pain	52.4 (23.9)	54.1 (22.6)	73.4 (20.2)	76.4 (19.0)
Symptoms	56.1 (15.6)	58.2 (21.1)	69.6 (21.8)	75.2 (19.7)
ADL	42.1 (22.2)	47.4 (20.6)	76.7 (20.2)	79.1 (18.0)
Sport/Rec	7.3 (25.9)	8.2 (23.3)	41.3 (30.7)	49.3 (32.6)
QOL	24.8 (15.4)	29.3 (19.2)	54.5 (23.0)	58.9 (24.7)

SD=standard deviation

At the follow-up six weeks after fracture, the effect sizes were small for all the KOOS subscales. At the 6- and 12-month follow-ups, large effect sizes were observed for all the KOOS subscales (See Table 3b).

**Table 3b:** Responsiveness

KOOS, ES	6 weeks	6 months	12 months
Pain	0.07	0.88	1.00
Symptoms	0.13	0.87	1.22
ADL	0.24	1.56	1.67
Sport/Rec	0.03	1.31	1.62
QOL	0.29	1.92	2.21

ES=effect size

The correlations between the change from 6 weeks and 6 moths in the KOOS subscales and the GROC at 6 months were: Symptoms 0.5, ADL 0.4, and QOL 0.4, in accordance with hyphothesis 1.

The correlations between the change from 6 months to 12 moths in the KOOS subscales and the GROC at 12 months were: ADL 0.4, Sport 0.5 and QOL 0.4, in accordance with hyphothesis 2.

The correlations between the change from 14 days and 6 weeks in the KOOS subscales and the GROC at 6 weeks were: Pain 0.4, Symptoms 0.4, in accordance with hyphothesis 3.

## Discussion

Results indicated that the KOOS questionnaire might be useful for measuring the trajectory of functional recovery and knee-related quality of life in patients following lateral tibial plateau fractures. The questionnaire showed acceptable relevance from a patient perspective, high reliability, and acceptable responsiveness. The present study lack information of content validity and information regarding minimal clinical important difference and more research is needed for further validation of psychometric properties of KOOS for patients with lateral tibial plateau fractures.

### Relevance of the KOOS items

Content validity was party evaluated by the present study by asking patients about the relevance of the 42 KOOS items [21]. To establish a full content validation information of comprehensiveness and comprehensibility and professionels rating are considered essential [21]. Such data was not part of the present study and may be included in future validation studies of the KOOS on lateral tibial plateau fractures.

The Knee Injury and Osteoarthritis Outcome Score (KOOS) was developed to assess the patient’s opinion about their knee and associated problems [18]. Adequate content validity has previously been reported for several knee complaints that can result in post-traumatic osteoarthritis and patients with osteoarthritis [16]. Despite the common use of the KOOS questionnaire in clinical practice and research on patients with lateral tibial plateau fractures, the content validity has not been established [1, 11–15]. The present study demonstrated a acceptable relevance for all the five KOOS subscales at the 12-month follow-up. At six weeks follow-up, the subscale Sport/Rec was reported with a low degree of relevance (mean 0.7). For the first six weeks, all patients were restricted with no weight bearing and equipped with braces; therefore, as expected, the subscale Sport/Rec was reported with a low degree of relevance. From a patient perspective results indicated that the KOOS questionnaire may be relevant for monitoring patients’ perceived outcomes following lateral tibial plateau fractures. Due to recommendation from the COSMIN framework a high validity is essential in recommending a PROM and more research may be needed to establish a full validation of the KOOS [21].

### Test-retest reliability

This study demonstrated the high test-retest reliability of 4 of the 5 KOOS subscales (Pain, ADL, Sport/Rec and QOL), with ICC values > 0.7. A single subscale (Symptoms) demonstrated moderate reliability (ICC = 0.6).

This study is the first to report on the reliability of the KOOS questionnaire for patients with lateral tibial plateau fractures. The results are comparable to those previously reported by other studies investigating test-retest reliability of KOOS subscales with patients presenting with knee injuries [16].

### Responsiveness

Mean KOOS subscale scores increase between 14 days and six weeks and at 6- and 12 months, indicating that the expected reduction in pain and increase in function during the first 12 months after a lateral tibial plateau fracture is captured by the KOOS questionnaire. The hyptheses tests of responsiveness showed moderate to high correlations between KOOS subscales and GROC throughout the follow-up period. Pre-defined hypotheses were established based on our clinical experience with treatment of patients with lateral tibial plateau fractures. During the first weeks following surgery pain and symptoms from the knee joint are common and later complaints from sport at other activities are predominant. Considering the difference between GROC and KOOS subscales moderate correlations were predefined and expected due to fundamental differences in the construct. Further analyses of effect size (ES) for all KOOS subscales showd low ES at 6 weeks. This result was expected as a bone union from lateral tibial plateau fractures is reported with an average of 14 weeks, indicating that several weeks following surgery may be needed to expect large improvement in the functional recovery [28]. At the 6- and 12-month follow-ups, large effect sizes were observed for all the KOOS subscales.

Previously, acceptable responsiveness of the KOOS have been reported for other orthopaedic conditions [16]. We believe that results reflect clinical practice and the expected recovery process of a lateral tibial plateau fracture well. Results indicating that the KOOS questionnaire demonstrated an acceptable responsiveness within one year following a lateral tibial plateau fracture.

### Limitations

The present study have several limitations. A major limitation of the present study may be the sample size of 41 patients. However, several studies reporting psychometric properties of the KOOS are available with comparable sample sizes [16].

Moreover, the present study lack in several recommendations from the COSMIN framework for evaluating measurement properties [21]. The COSMIN framework is a consensus-based and highly acknowledged framework that covers measurement properties definitions and a methodological approach for evaluation [21]. As a consequence more research is needed to validate KOOS to patients with tibial plateau fractures. Furthermore, the generalizability of the study may be limited due to the limited sample size, however the present study included a consecutive serie of patients and the study is the first to provide psykometric properties of the widely used KOOS questionnaire to patients with lateral tibial plateau fractures. Moreover, the loss of follow-up may be a limitation, however this is comparable to other cohort studies including patients with lateral tibial plateau fractures [5].

## Conclusion

The KOOS questionnaire showed acceptable relevance, high test-retest reliability and acceptable responsiveness within one year following a lateral tibial plateau fracture. More research is needed for further validation of psychometric properties of KOOS for patients with lateral tibial plateau fractures.

## Data Availability

No datasets were generated or analysed during the current study.
